# Stroke and Bleeding Risk Assessments in Patients With Atrial Fibrillation: Concepts and Controversies

**DOI:** 10.3389/fmed.2020.00054

**Published:** 2020-02-21

**Authors:** Wern Yew Ding, Stephanie Harrison, Dhiraj Gupta, Gregory Y. H. Lip, Deirdre A. Lane

**Affiliations:** ^1^Liverpool Centre for Cardiovascular Science, Liverpool Heart and Chest Hospital, University of Liverpool, Liverpool, United Kingdom; ^2^Aalborg Thrombosis Research Unit, Department of Clinical Medicine, Aalborg University, Aalborg, Denmark

**Keywords:** stroke, bleeding, risk factors, risk assessment, atrial fibrillation

## Abstract

Risk assessments are an important element in the management of patients with atrial fibrillation (AF). In this review, we aim to discuss the concepts and controversies surrounding the various risk factors for stroke and bleeding in AF. Indeed, there are a variety of clinical, electrical, biological, and genetic markers to guide stroke and bleeding risk assessments in AF. The more common factors have been used to formulate risk stratification scores. Some risk factors have shown promise, but others remain less well-defined. Our aim is to discuss concepts and controversies surrounding current evidence of risk factors for stroke and bleeding assessments in AF.

## Introduction

Risk assessments are an important element in clinical practice. Therefore, it is crucial to understand the evidence supporting the individual risk factors to help guide management of patients with complex conditions such as atrial fibrillation (AF). Atrial fibrillation is the most common sustained cardiac arrhythmia. In 2010, the estimated global prevalence of AF was 33.5 million with approximately 5 million new cases identified (1). The condition is associated with a greater risk of stroke and heart failure, reduced quality of life, and a 2-fold increased mortality (1–5). In addition, it poses a significant healthcare economic burden. Conservative estimates in the United Kingdom found that AF was associated with a direct annual cost of £459 million, based on an estimated 0.5 million affected patients (6). However, as AF has detrimental effects on many other comorbidities, the total cost of AF is expected to be significantly higher. A study in the United States on AF-related cost estimates this to be as high as $26 billion per year (7). Perhaps most worryingly, the incidence and prevalence of AF is increasing worldwide (8, 9). Between 5.6 and 15.9 million people in the United States are projected to have AF by 2050 (10, 11) and 17.9 million people in Europe by 2060 (12).

Given the higher stroke risk associated with AF, an integrated approach in the management of patients with AF must include accurate stroke risk stratification. Patients who are *not* identified as “low risk” should then be offered anticoagulation therapy to reduce their risk of stroke. However, the use of anticoagulation carries an increased risk of bleeding. Most bleeding events are multifactorial in nature and some may have devastating clinical consequences. Therefore, it is important to consider and treat any modifiable bleeding risk factors prior to commencing anticoagulation.

Overall, stroke and bleeding risk assessments in AF are complex with ever emerging evidence. Therefore, it can be challenging for clinicians to stay up to date with the most recent literature and appreciate the interplay of the various factors involved. This article is not an exhaustive systematic review of the vast literature on this topic. Our aim is to discuss concepts and controversies surrounding current evidence of risk factors for stroke and bleeding assessments in AF.

## Stroke Risk Assessment

**Figure 1:**
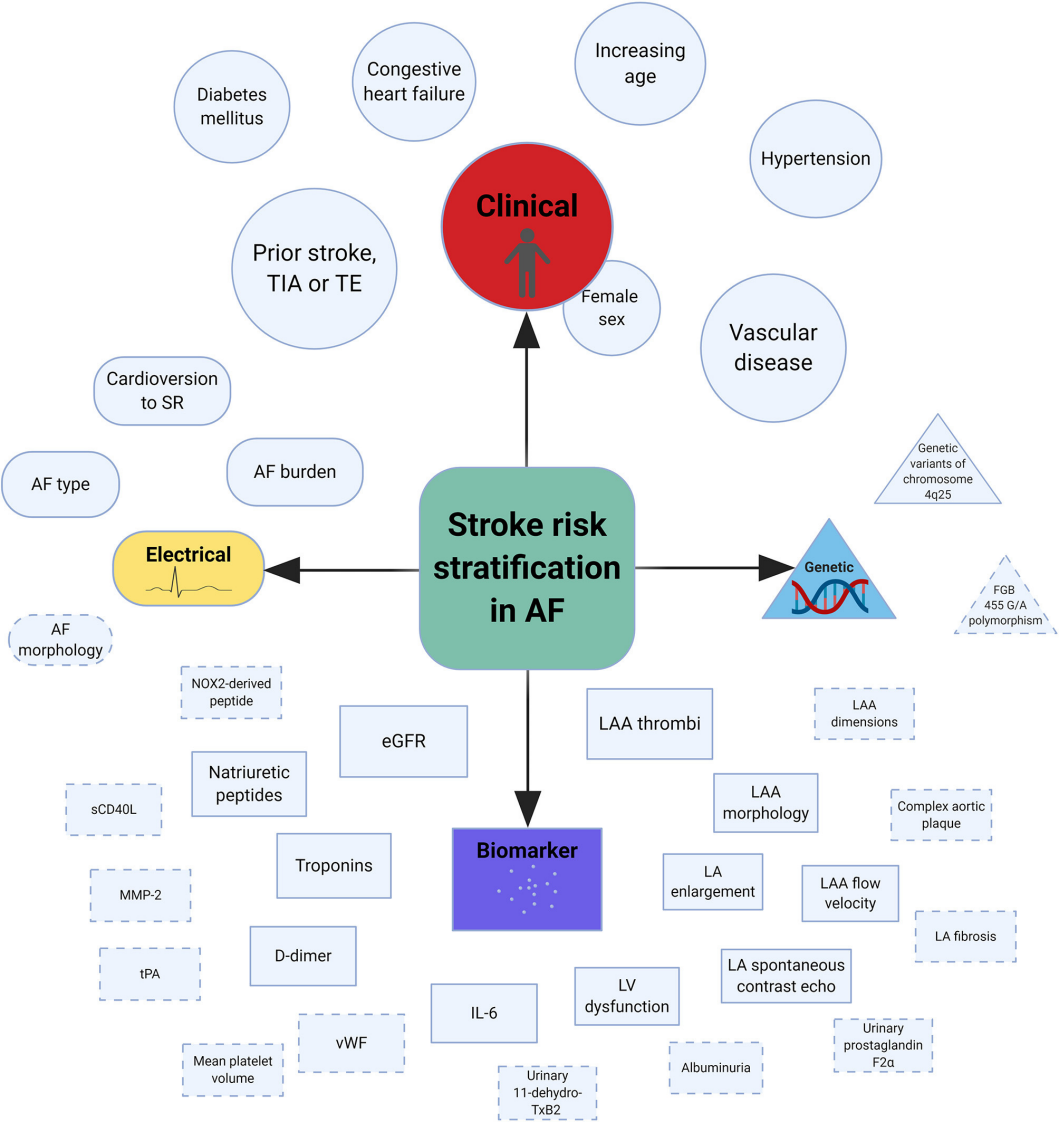
Central illustration of risk factors associated with stroke in AF. AF, atrial fibrillation; eGFR, estimated glomerular filtration rate; IL-6, interleukin-6; LA, left atrial; LAA, left atrial appendage; LV, left ventricle; MMP-2, matrix metalloproteinase-2; NOX2, reduced nicotinamide adenine dinucleotide phosphate oxidase 2; sCD40L, soluble CD40 ligand; SR, sinus rhythm; TE, thromboembolism; TIA, transient ischemic attack; tPA, tissue plasminogen activator; vWF, von Willebrand factor. *Dashed lines indicate possible risk factors and the size of individual shapes reflect the degree of risk (Created with BioRender).

In general, AF is associated with a 5-fold increased risk of stroke (13). Furthermore, stroke outcomes are more severe in the presence of AF, as determined by clinical or radiological assessment (14, 15). Various factors based on clinical, electrical, biological, and genetic markers have been shown to predict stroke risk in AF (Table 1, Figure 1). Using a culmination of different risk factors, predominantly clinical, various authors have developed a total of at least 15 risk scores to assist stroke risk stratification in AF (16–19).

**Table 1:** Risk factors for stroke in AF.

	**Risk factor**	**Possible risk factor**
Clinical markers	Prior stroke, TIA or TE Vascular disease^+^ Increasing age Congestive heart failure Hypertension Diabetes mellitus Female sex^*^	
Electrical markers	AF burden Cardioversion to SR AF type	AF morphology
**BIOLOGICAL MARKERS**		
Blood markers	Troponins I and T BNP and NT-proBNP Reduced eGFR D-dimer Interleukin-6	von Willebrand factor Mean platelet volume MMP-2 NOX2-derived peptide Soluble CD40 ligand Tumor necrosis factor-α tPA β-thromboglobulin
Urine markers		Albuminuria Prostaglandin F_2α_ 11-dehydro-thromboxane B2
Imaging markers	LAA thrombi LA spontaneous echo contrast LAA flow velocity LAA morphology LV dysfunction LA enlargement	LA fibrosis LAA dimensions Complex aortic plaque
Genetic marker	Genetic variants on chromosome 4q25	FGB 455 G/A polymorphism

AF, atrial fibrillation; BNP, B-type natriuretic peptide; eGFR, estimated glomerular filtration rate; LA, left atrial; LAA, left atrial appendage; LV, left ventricle; MMP-2, matrix metalloproteinase-2; NOX2, reduced nicotinamide adenine dinucleotide phosphate oxidase 2; NT-proBNP, N-terminal pro-B-type natriuretic peptide; SR, sinus rhythm; TE, thromboembolism; TIA, transient ischemic attack; tPA, tissue plasminogen activator.

* Risk modifier.

+ *Includes prior myocardial infarction, peripheral artery disease, or aortic plaque*.

At present, the majority of clinical guidelines recommend the use of CHA_2_DS_2_-VASc score to aid decision on anticoagulation therapy (20–24). This tool was refined from the original 2006 Birmingham “National Institute for Health and Care Excellence” stroke risk schema with a specific focus on optimal identification of low-risk individuals and subsequently validated in several large AF cohorts (18, 25). In general, higher scores are associated with an increased annual risk of ischemic stroke. Males with a score of 0 and females with a score of 1 are considered at “low-risk” of stroke, with event rates <1% per year. Current recommendations support consideration of anticoagulation therapy in all other patients where there is sufficient stroke risk to justify the hazards of anticoagulation.

Older studies have suggested that so-called “lone AF,” contributes to an annual stroke risk of 1% (26). However, more contemporary studies have found that stroke rate in such low-risk patients is <1% per year, as defined by age <65 years and the absence of any established stroke risk factors (27).

### Clinical Markers

Atrial fibrillation is a multi-systemic disorder that often occurs alongside other comorbidities. Many of these co-morbidities are risk factors for incident AF and may also increase the risk of subsequent complications. Pooled analysis from five randomized controlled trials (RCTs) demonstrated that a history of stroke or transient ischemic attack (TIA), increasing age, hypertension, diabetes mellitus, and congestive heart failure were individual risk factors for stroke in AF (26). A more recent systematic review of seven studies which included over 12,000 patients found similar results, although there was inconclusive evidence to support congestive heart failure and coronary artery disease as risk factors (28). Prior stroke or TIA was the most powerful predictor of further stroke events, contributing to an annual risk of >5%. Increasing age (per decade), hypertension and diabetes mellitus were each associated with a 1.5–2-fold greater stroke risk. In a separate study, Olesen et al. demonstrated that the presence of heart failure, previous stroke, and vascular disease were independent predictors of stroke or thromboembolism among AF patients aged under 65 years (29). A Swedish cohort study found that peripheral artery disease, vascular disease, prior myocardial infarction, female sex, prior embolism, intra-cranial hemorrhage (ICH), hypertension, and diabetes mellitus were associated with additional thromboembolic events (25). The mechanism by which the conditions above influence stroke risk in AF is likely multifactorial and partly related to its influence on progression of the disease through atrial substrate remodeling. Furthermore, many of these conditions are pro-thrombotic by nature.

Among non-anticoagulated patients in the ATRIA study, females had a 60% higher risk of thromboembolism compared to males (30). Similar findings were shown in a cohort of anticoagulated patients where females had a 2-fold higher risk of ischemic stroke (31). However, a subsequent population-based cohort study of 147,622 patients with AF failed to reproduce these results (32). A study by Nielsen et al. demonstrated similar rates of thromboembolism for both sexes among AF patients deemed at lowest risk (33). Excess risk in females were only evident for those with two or more non-sex-related stroke risk factors. Therefore, although female sex should remain an important component for stroke risk assessment in AF, it should be considered in the context of other risk factors (“risk modifier”).

### Electrical Markers

Several electrical markers relating to AF have been described to predict stroke risk (Table 2). The impact of AF type (paroxysmal or sustained) on stroke risk remains controversial with earlier studies reporting a similar risk of stroke and systemic thromboembolism in paroxysmal AF compared to sustained or permanent AF (39, 41, 44). These results were supported by two independent systematic reviews which included a total of nine relevant studies (28, 45). However, a third systematic review of 12 studies which included just under 100,000 patients demonstrated that non-paroxysmal AF was associated with a hazard ratio of 1.38 [95% confidence interval (CI), 1.17–1.57] for thromboembolism compared to paroxysmal AF, after multivariable adjustment (46). The finding was reinforced by results from the ENGAGE AF-TIMI 48 trial which showed fewer thromboembolic events among patients with paroxysmal AF compared to those with sustained AF (36). There was no difference in thromboembolic risk between patients with persistent and permanent AF in the study. Given the current evidence, it would appear that sustained AF is likely to be associated with a higher stroke risk overall. However, it remains unclear whether the increased risk is due to shared underlying mechanisms of the disease or if the burden of AF itself is directly implicated.

**Table 2:** Electrical markers relating to AF and stroke risk.

**References**	**Design**	**Study population**	***n***	**% male; mean (SD) or median (IQR) age, years**	**Relevant outcome measures**	**Follow-up duration**	**Electrical marker**	**Findings**
Chu et al. (34)	Retrospective cohort	Undergoing pacemaker implantation	152	56.4; 73.2 (13.3)	Ischemic stroke, TIA or SE	67 months	AF burden	Higher AF burden was associated with greater risk of ischemic stroke, TIA, or SE
Go et al. (35)	Retrospective cohort	AF on 14-day ambulatory ECG monitoring	1,965	55.2; 68.8 (11.8)	Ischemic stroke and SE	NA (retrospective)	AF burden	Higher AF burden was associated with greater risk of ischemic stroke or SE
Link et al. (36)	Sub-analysis of RCT	AF with at least 2 stroke risk factors	21,105	61.9; 70.6 (NA)	Stroke or SE	2.8 years	AF type	Paroxysmal AF was associated with lower risk of stroke or SE compared to persistent and permanent AF
Boriani et al. (37)	Pooled analysis of 5 prospective studies	Pacemaker or ICD *in situ*	10,016	69; 70 (61–76)	Ischemic stroke	24 months	AF burden	Higher AF burden was associated with greater risk of ischemic stroke
Healey et al. (38)	RCT	Aged ≥65 years, known HTN and recent pacemaker or ICD implanatation	2,580	58.4; 76.1 (NA)	Ischemic stroke or SE	2.5 years	AHRE (>190 bpm for >6 min)	Presence of AHREs were associated with a 5.6-fold greater risk of ischemic stroke or SE
Friberg et al. (39)	Retrospective cohort	AF (or atrial flutter)	1,981	NA; 75.8 (NA)	Ischemic stroke	3.6 years	AF type	No association with Ischemic stroke
Yilmaz et al. (40)	Retrospective cohort	AF lasting longer than 24 h	811	45.9; 60 (13)	History of stroke or TIA	NA (retrospective)	Coarse or fine AF	Coarse AF was associated with greater risk of stroke or TIA
Hohnloser et al. (41)	RCT	AF with at least 1 stroke risk factor	6,706	66; 70.2 (NA)	Stroke or SE	1.3 years	AF type	No association with stroke or SE
Capucci et al. (42)	Prospective cohort	Indication for pacemaker and history of symptomatic atrial tachyarrhythmias	725	49.7; 71 (11)	Ischemic stroke, TIA or SE	22 months	AF duration	AF duration longer than 24 h was associated with greater risk of ischemic stroke, TIA, or SE
Glotzer et al. (43)	RCT	SND undergoing pacemaker implantation	312	45; 74 (NA)	Death of non-fatal stroke	33.1 months	AHRE (>220 bpm for 10 consecutive beats)	Presence of AHREs were associated with greater risk of composite endpoint
Hart et al. (44)	Prospective cohort	AF	2,012	71.5; 69.1 (NA)	Ischemic stroke	2 years	AF type	No association with ischemic stroke

*AF, atrial fibrillation; AHRE, atrial high rate episode; ECG, electrocardiogram; HTN, hypertension; ICD, implantable cardioverter defibrillator; IQR, interquartile range; NA, not applicable or available; RCT, randomized controlled trial; SD, standard deviation; SE, systemic embolism; SND, sinus node disease; TIA, transient ischemic attack*.

The method(s) by which AF is identified has evolved significantly over the past decade. While AF was previously detected predominantly using standard 12-lead electrocardiogram (ECG), the increased use of implantable devices has contributed to a rise in “device-detected AF.” The rise of device-detected AF has also led to new terms such as “atrial high-rate episodes” (AHRE) and “subclinical atrial tachyarrhythmias”—both of which are sometimes used interchangeably with AF. These changes have sparked important questions such as “what duration of AF is required for diagnosis?”, “what is the threshold of AF burden where it becomes clinically important?” and “what is the threshold of AF burden at which anticoagulation will provide a net benefit?” While many of these questions remain unanswered, there are some evidence to shed light on the matter. Several studies have investigated the threshold of AF burden associated with a negative clinical outcome. The MOST study found that patients with at least one AHRE (defined as an atrial rate >220 bpm) lasting ≥5 min had a 2-fold risk of stroke or mortality, and 6-fold risk of developing AF compared to patients without AHRE (43). The ASSERT study similarly found that subclinical atrial tachyarrhythmias (defined as an atrial rate >190 bpm) lasting >6 min was associated with an increased risk of incident AF, and ischemic stroke or systemic embolism (38). In contrast, Capucci et al. performed a study using pre-specified AF durations in which the authors demonstrated that AF duration of >5 min was not associated with thromboembolic events unlike episodes >24 h which were independently associated with thromboembolic events (42). A potential explanation for the differences observed in this study may be related to the fact that a significant proportion of patients had short episodes of AF (about 80%). Pooled analysis of five prospective studies that included 10,016 patients with implantable devices found that AF burden was an independent predictor of ischemic stroke (37). In this study, patients with 1 h of AF per day were found to be at highest risk. Among patients with paroxysmal AF, authors of the KP-RHYTHM study reported a 3-fold increased risk of thromboembolism in those with the highest tertile of AF burden (≥11.4%) compared to the lower two tertiles, after adjusting for either ATRIA or CHA_2_DS_2_-VASc score (35). A further study in patients with dual-chamber pacemakers also confirmed that patients with thromboembolism had higher AF burden (34). Overall, AF burden may reflect the proportion of time spent in mechanical dyssynchrony, thereby promoting thrombus formation. Though it is recognized to be an important predictor of stroke risk, the exact relationship remains to be defined and further research is warranted. Furthermore, determining the AF burden in patients without implantable cardiac devices can be challenging.

There are also important considerations when treating patients with a high burden of AF. Although a strategy to reduce this burden may be appropriate for some patients, attempts to restore sinus rhythm is not without risk. The FibStroke study revealed that electrical and pharmacological cardioversions were related to occurrences of ischemic stroke (47). As the majority of events occurred in patients who underwent electrical cardioversion, it could be postulated that the delivery of electrical energy may have dislodged pre-formed thrombi. However, many of the stroke events only occurred after a significant time delay (median of 2 days) following cardioversion. Therefore, there are likely to be other factors involved. Furthermore, there are even reports of acute thromboembolic complications following spontaneous cardioversion of AF (48, 49). A potential cause for this may be linked to atrial stunning that occurs regardless of the means of cardioversion (50).

It was previously suggested that the morphology of AF as assessed on ECG may be useful for stroke risk assessment. In a study of 811 consecutive patients, Yilmaz et al. classified AF based on surface ECG as “coarse” or “fine” AF, and was able to demonstrate that patients with coarse AF had increased risk of stroke (40). The authors defined “coarse” AF as the presence of undulations moving ≥1 mm from the isoelectric baseline with different morphologies and “fine” AF as the presence of minimal or no undulation from the isoelectric baseline. At present, there is insufficient evidence to draw any firm conclusions. Nevertheless, if deemed reliable, classification of AF according to the different morphologies on ECG may provide a readily assessible tool to support clinical decisions.

Given the increased risk of stroke in AF, it would seem plausible that a temporal relationship might exists between these two conditions. If true, it may provide us a method of identifying patients at the point of highest stroke risk in order to instigate additional protective measures to avoid this complication. However, an initial study by ASSERT investigators revealed that only 8% of patients had subclinical AF detected within 30 days before their stroke or systemic embolism (51). Thus far, there is no strong evidence to support a temporal relationship between the episodes of AF and stroke events.

### Biological Markers (“Biomarkers”)

Many biomarkers involving blood, urine, and structural parameters have been studied in AF and been shown to improve the accuracy of stroke risk stratification. Despite this, their clinical applicability remains limited. Possible reasons include inter- and intra-patient and assay variability; diurnal variation of the results obtained; costs involved; strong influences of associated comorbidities and treatments in AF on these parameters; and lack of specificity. As such, these biomarkers are mainly reserved for research purposes.

#### Blood-Based Biomarkers

In general, blood-based biomarkers may be divided into those that relate to cardiac function (troponins and natriuretic peptides), hemostatic processes [D-dimer, von Willebrand factor (vWF), soluble E-selectin, and P-selectin], inflammation [interleukin-6 (IL-6) and C-reactive protein (CRP)], or “others” (renal function) (Table 3).

**Table 3:** Blood-based biomarkers for stroke risk in AF.

**References**	**Design**	**Study population**	***n***	**% male; mean (SD) or median (IQR) age, years**	**Relevant outcome measures**	**Follow-up duration**	**Biomarker**	**Findings**
Park et al. (52)	Prospective registry	AF	10,978	63.6; 73.5 (11.8)	Stroke	42.6 months	Platelet count (<100 × 10^9^/L)	Lower platelet counts were associated with lower risk of stroke
Janion-Sadowska et al. (53)	Prospective cohort	AF on NOAC	124	33.1; 70.3 (NA)	Stroke or TIA	55 months	Platelet count (<100 × 10^9^/L)	No association with stroke or TIA
Rivera-Caravaca et al. (54)	Prospective cohort	AF on OAC, attending clinic	1,226	49.7; 76 (70–81)	Ischemic stroke	6.5 years	Soluble fibrin monomer complex	No association with ischemic stroke
You and Tang (55)	Case-controlled study	Non-anticoagulated AF	323	63.8; 75.2 (NA)	Ischemic stroke	NA (retrospective)	D-dimer	No association with ischemic stroke
Ancedy et al. (56)	Prospective cohort	Hospitalized AF	122	46; 70 (14)	Composite of all-cause death and stroke; stroke	5 years	vWF	Higher vWF levels were associated with greater risk of composite endpoint No association with stroke risk only
Hayashi et al. (57)	Prospective registry	AF	1,013	71.6; 72.8 (9.7)	Stroke, TIA, or SE	25 months	BNP	High BNP levels were associated with a 3.9-fold greater risk of stroke, TIA, or SE
Choi et al. (58)	Prospective cohort	AF	352	57.4; 68.4 (12.1)	Composite of ischemic stroke and incidental LA thrombus	35.4 months	Antithrombin III	No association with composite endpoint
							MPV	High MPV levels were associated with a 6.4-fold greater risk of composite endpoint
Aulin et al. (59)	Sub-study of RCT	AF with at least 1 stroke risk factor	6,187	63.7; 72 (67–77)	Stroke or SE	2 years	IL-6	Higher IL-6 levels were associated with greater risk of stroke or SE
							CRP	No association with stroke or SE
							Fibrinogen	No association with stroke or SE
Pignatelli et al. (60)	Prospective cohort	AF	950	55.5; 73.3 (8.8)	Composite of stroke, TIA, MI, and coronary revascularization	25.7 months	Serum NOX2-derived peptide	Higher serum NOX2-derived peptide levels were associated with greater risk of composite endpoint
Banerjee et al. (61)	Prospective cohort	AF	5,912	62.9; 70.9 (NA)	Ischemic stroke or TE	2.5 years	eGFR (MDRD)	Lower levels of renal function were associated with greater risk of ischemic stroke or TE
Roldan et al. (62)	Prospective cohort	AF on OAC, attending clinic	1,172	49; 76 (71–81)	Stroke or TIA	34 months	NT-proBNP	High NT-proBNP levels were associated with a 2.7-fold greater stroke or TIA risk
Apostolakis et al. (63)	*Post-hoc* analysis of RCT	AF	4,576	66.5; 70 (9)	Stroke or SE	10.8 months	CrCl, eGFR (MDRD, CKD-EPI)	Lower levels of renal function were associated with greater risk of stroke or SE
Krishnamoorthy et al. (64)	Prospective cohort	AF, attending clinic	423	55.6; 72.7 (8.4)	Composite of stroke, acute MI, and all-cause mortality; Ischemic stroke	19 months	vWF	Higher vWF levels were associated with greater risk of composite endpoint and Ischemic stroke
							Soluble E-selectin	Higher soluble E-selectin levels were associated with greater risk of composite endpoint and Ischemic stroke
Hijazi et al. (65)	Sub-study of RCT	AF with at least 1 CHADS_2_ risk factor	14,892	64.4; NA	Stroke or SE	1.9 years	NT-proBNP	Higher NT-proBNP levels were associated with greater risk of stroke or SE
								Highest quartile of NT-proBNP was associated with 2.4-fold greater risk of stroke or SE compared to lowest quartile
Piccini et al. (17)	Sub-study of RCT	AF with at least 1 stroke risk factor	14,264	60.7; 73 (NA)	Stroke or SE	1.9 years	CrCl, eGFR (MDRD)	Lower levels of renal function were associated with greater risk of stroke or SE; every 10-mL/min decrease in CrCl resulted in 1.12-fold increase in risk; every 5 mL/min/1.73 m^2^ decrease in eGFR (MDRD) resulted in 1.09-fold increase in risk
Hijazi et al. (66)	Sub-study of RCT	AF with at least 1 stroke risk factor	6,189	63.7; 72 (67–77)	Stroke	2.2 years	NT-proBNP	Higher NT-proBNP levels were associated with greater stroke risk Highest quartile of NT-proBNP was associated with 2.4-fold greater risk of stroke compared to lowest quartile
							Troponin I	Higher troponin I levels were associated with greater stroke risk Highest quartile of troponin I was associated with 2.0-fold greater risk of stroke compared to lowest quartile
Roldan et al. (67)	Prospective cohort	AF on OAC, attending clinic	930	51; 76 (70–81)	Stroke or TIA	2 years	Troponin T	High troponin T levels were associated with a 2.4-fold greater stroke or TIA risk
							IL-6	No association with stroke or TIA
Ehrlich et al. (68)	Prospective cohort	AF	278	63; 70 (11)	Composite of stroke, MI, SE, and all-cause death	28 months	hsCRP	No association with composite endpoint
							sCD40L	No association with composite endpoint
							MMP-2	Higher MMP-2 levels were associated with greater risk of composite endpoint
							vWF	No association with composite endpoint
							sVCAM-1	Higher sVCAM-1 levels were associated with greater risk of composite endpoint
Roldan et al. (69)	Prospective cohort	AF on OAC, attending clinic	829	50; 76 (70–80)	Composite of TE, acute MI, and acute HF	27.6 months	vWF	High vWF levels were associated with a greater risk of composite endpoint
Ha et al. (70)	Prospective cohort	AF	200	56; 68.9 (11.7)	Ischemic stroke	15.1 months	MPV	Higher MPV levels were associated with greater ischemic stroke risk Highest tertile of MPV was associated with a 5.0-fold greater risk of ischemic stroke compared to lowest quartile
Sadanaga et al. (71)	*Post-hoc* analysis of prospective cohort	AF on OAC	261	56; 74 (9)	Ischemic stroke, TIA, or SE	24.7 months	BNP	High BNP levels were associated with a 5.3-fold greater risk of ischemic stroke, TIA, or SE
Sadanaga et al. (72)	Prospective cohort	AF on OAC	269	57; 74 (9)	Ischemic stroke, TIA, or SE	25.2 months	D-dimer	High D-dimer levels were associated with a 15.8-fold greater risk of ischemic stroke, TIA, or SE
Go et al. (73)	Sub-study of prospective cohort	AF	10,908	57.2; 71.6 (NA)	TE	3 years	eGFR (MDRD)	Lower levels of renal function were associated with greater TE risk
Pinto et al. (74)	Prospective cohort	Chronic AF	373	63.5; 66.1 (7.4)	Ischemic stroke	3 years	IL-1β	No association with ischemic stroke
							TNF-α	Higher TNF-α levels were associated with greater ischemic stroke risk
							IL-6	Higher IL-6 levels were associated with greater ischemic stroke risk
							IL-10	No association with ischemic stroke
							E-selectin	No association with ischemic stroke
							P-selectin	No association with ischemic stroke
							ICAM-1	No association with ischemic stroke
							VCAM-1	No association with ischemic stroke
							vWF	Higher vWF levels were associated with greater ischemic stroke risk
Ferro et al. (75)	Prospective cohort	AF	231	48; 72.4 (10.3)	Composite of stroke and MI	27.8 months	sCD40L	High sCD40L levels were associated with a 4.6-fold greater risk of composite endpoint
Nozawa et al. (76)	Prospective cohort	AF	509	64.8; 66.6 (10.3)	Composite of clinically evident stroke, TIA, and SE	2 years	D-dimer	High D-dimer levels were associated with a greater risk of composite endpoint
							F1+2	No association with composite endpoint
							Platelet factor 4	No association with composite endpoint
							β-thromboglobulin	No association with composite endpoint
Conway et al. (77)	Prospective cohort	AF, attending clinic	77	57; 68 (62–75)	Stroke	6.3 years	IL-6	High IL-6 levels were associated with a 2.9-fold greater stroke risk
							CRP	No association with stroke
Vene et al. (78)	Prospective cohort	AF referred to clinic for initiation of OAC	113	60; 70.2 (5.4)	Composite of stroke, MI, SE, peripheral vascular occlusion, and cardiovascular death	44.3 months	D-dimer	Higher D-dimer levels were associated with greater risk of composite endpoint
							tPA	Higher tPA levels were associated with greater risk of composite endpoint
							F1+2	No association with composite endpoint
							TAT complexes	No association with composite endpoint
							PAI-1	No association with composite endpoint
Feinberg et al. (79)	Sub-study of prospective cohort	AF with at least 1 high-risk stroke factor	1,531	NA; 70 (NA)	Ischemic stroke or SE	2 years	F1+2	No association with ischemic stroke or SE
							β-thromboglobulin	No association with ischemic stroke or SE
							Fibrinogen	No association with ischemic stroke or SE
							Factor V Leiden mutation	No association with ischemic stroke or SE

*AF, atrial fibrillation; BNP, B-type natriuretic peptide; CrCl, creatinine clearance; CRP, C-reactive protein; eGFR, estimated glomerular filtration rate; F1+2, prothrombin fragment F1+2; hsCRP, high-sensitivity C-reactive protein; ICAM-1, intercellular adhesion molecule-1; IL-10, interleukin-10; IL-1β, interleukin-1β; IL-6, interleukin-6; IQR, interquartile range; LA, left atrial; MDRD, Modification of Diet in Renal Disease Study; MI, myocardial infarction; MMP-2, matrix metalloproteinase-2; MPV, mean platelet volume; NA, not applicable or available; NOAC, non-vitamin K oral anticoagulants; NOX2, reduced nicotinamide adenine dinucleotide phosphate oxidase 2; NT-proBNP, N-terminal pro-B-type natriuretic peptide; OAC, oral anticoagulation; PAI-1, plasminogen activator inhibitor-1; RCT, randomized controlled trial; sCD40L, soluble CD40 ligand; SD, standard deviation; SE, systemic embolism; sVCAM-1, soluble vascular cell adhesion molecule-1; TAT, thrombin-antithrombin; TE, thromboembolism; TIA, transient ischemic attack; TNF-α, tumor necrosis factor-alpha; tPA, tissue plasminogen activator; vWF, von Willebrand factor*.

##### Cardiac function

Troponins and natriuretic peptides are among the most frequently used cardiac biomarkers. Their value in a variety of cardiovascular diseases such as myocardial infarction and heart failure have previously been established (80, 81). Further studies have also consistently demonstrated that levels of these biomarkers may be used to improve predictions of stroke risk in AF (57, 62, 65–67, 71). A RE-LY sub-study found that elevations in troponin I and N-terminal pro-B-type natriuretic peptide (NT-proBNP) were common among AF patients (66). Both were independently related to an increased risk of stroke and there was a graded relationship such that patients with higher levels of these cardiac biomarkers were at greater risk compared to those with lower levels. The highest quartile of NT-proBNP was associated with 2.4-fold greater risk of stroke compared to the lowest quartile while the higher tertile of troponin I was associated with a 2.0-fold greater risk of stroke compared to the lowest tertile. There are several proposed mechanisms for the prognostic value of these cardiac biomarkers. In AF, unlike heart failure, B-type natriuretic peptides (BNPs) may originate from the atria (82). This is supported by the fact that restoration of sinus rhythm is associated with a rapid fall in the level of natriuretic peptides (83, 84). The elevated levels of natriuretic peptides may reflect the degree of atrial stretch (85). This in turn indicates atrial dysfunction which is linked to thrombus formation (86). Meanwhile, troponins are released as a following myocardial injury which may promote a pro-thrombotic state. Furthermore, elevated levels of troponin has been associated with impaired left atrial function, as assessed by cardiac magnetic resonance imaging (MRI) (87).

##### Renal function

Another important biomarker in stroke risk stratification is renal function (17, 61, 63, 73). Impaired renal function was demonstrated to be a strong predictor of stroke and systemic embolism in the ROCKET AF and ATRIA study cohorts, second only to prior stroke or TIA (17). A meta-analysis of 18 studies involving 538,479 patients with AF demonstrated that estimated glomerular filtration rate (eGFR) was an independent risk factor for stroke or systemic embolism, with worsening chronic kidney disease (CKD) being associated with a greater increased risk (88). Indeed AF patients with the most severe form of CKD requiring dialysis may have a dramatic increase of 9.8-fold in stroke risk (89). Chronic kidney disease promotes a pro-thrombotic state by its effects on the individual components of Virchow's triad (90). It has been found to be associated with stasis of the left atrium (LA) (91, 92), impaired endothelial function (93–97) and enhanced platelet activation (98, 99). Furthermore, CKD is linked to the release of procoagulant and inflammatory biomarkers (99–101).

It was previously suggested that the inclusion of CKD as a risk factor may improve stroke prediction models (17). However, additional studies have found that it did not improve the discriminative capabilities of the CHADS_2_ and CHA_2_DS_2_-VASc scores (63, 102, 103). To summarize prior results, a meta-analysis of eight studies found that the inclusion of CKD resulted in a slight improvement for stroke prediction by the CHADS_2_ score but not with the CHA_2_DS_2_-VASc score (88). Therefore, there is currently insufficient evidence to justify the addition of CKD to the guideline-recommended CHA_2_DS_2_-VASc score. This is perhaps unsurprising given that CKD is associated with the individual component risk factors within the CHA_2_DS_2_-VASc score.

##### Hemostasis

Stroke risk in AF is strongly related to the disruption of hemostasis, leading to a pro-thrombotic state. However, the hemostatic processes are complex and involve many different pathways. Therefore, it is important to understand which of these are affected in AF. D-dimer is a small protein fragment that is released following fibrinolysis. A prospective study of 509 patients with AF, found that those with a D-dimer level of <150 ng/ml had significantly lower risk of thromboembolic events compared to those with D-dimer level of ≥150 ng/ml, 0.7% per year compared to 3.8% per year (76). Similar findings were demonstrated in other studies (72, 78). In contrast, You et al. reported that D-dimer was not an independent risk factor for ischemic stroke in AF despite finding a positive correlation between D-dimer levels and stroke risk scores (CHADS_2_ and CHA_2_DS_2_-VASc) (55). However, this study was retrospective in nature and only included non-anticoagulated patients. Overall, it does appear that D-dimer may be helpful for stroke risk stratification in AF.

Given the role of platelets in hemostasis, it would seem likely that platelet count may be associated with stroke risk. However, in a study of 124 patients with AF on non-vitamin K oral anticoagulants (NOAC), Janion-Sadowska et al. found no association between thrombocytopenia (platelet count <100 × 10^9^/L) and the risk of stroke or TIA over a 55-month follow-up period (53). In contrast, Park et al. recently reported retrospective registry data on 10,978 patients with AF where patients with a platelet count <100 × 10^9^/L had a significantly lower stroke risk compared to those with a normal platelet count (52). A major difference between the trials was in terms of the use of anticoagulation. About half of the patients (55.4%) in the latter trial were not anticoagulated and among those who were, warfarin was the main agent of choice (96.8%). There is limited evidence to base any firm conclusions at present although it could be that thrombocytopenia is protective against stroke in AF.

Von Willebrand factor is a glycoprotein integral to hemostasis. Raised levels of vWF has been associated with a pro-thrombotic state in AF (56, 64, 69, 74). However, a limitation in many of these studies was that the primary outcome measure included events such as heart failure and all-cause death. Therefore, it was difficult to draw strong conclusions from them. Among those that evaluated stroke only outcomes, two studies found that high levels of vWF was linked to a greater risk of stroke (64, 74). Despite demonstrating that higher levels of vWF were associated with a greater composite risk of all-cause death and stroke, Ancedy et al. found that the results were not significant when evaluated for stroke events only (56). Consequently, the role of vWF for stroke risk stratification in AF requires additional investigation.

##### Inflammation

There is ample evidence to support the importance of inflammation in AF. However, use of inflammatory biomarkers to predict stroke risk in this condition has been met with conflicting results. An early pilot study demonstrated that IL-6 was an independent predictor of stroke risk in AF, but not CRP (77). Subsequently, Pinto et al. evaluated this by comparing plasma levels of interleukin-1β, tumor necrosis factor-alpha, IL-6 and interleukin-10 (and E-selectin, P-selectin, intercellular adhesion molecule-1, vascular cell adhesion molecule-1 and vWF) in chronic AF patients with and without new-onset ischemic stroke over a period of 3 years (74). Following multivariable adjustment, only IL-6 and tumor necrosis factor-alpha remained significant predictors of stroke risk. In a separate study, Aulin et al. postulated that levels of inflammatory markers (IL-6, CRP, and fibrinogen) may be related to the risk of thromboembolism in AF (59). After adjustment for clinical risk factors, only IL-6 was found to be significant. However, use of other biomarkers (troponin, NT-proBNP, and cystatin-C) attenuated the importance of IL-6 such that it was no longer predictive of stroke risk in AF. Other studies failed to demonstrate an association between thromboembolic risk in AF and levels of high-sensitivity CRP (68), IL-6 (67), or fibrinogen (79). Given the current evidence, it appears that high levels of underlying inflammation as detected by IL-6 indicates a pro-thrombotic state in AF. There is no indication that CRP or fibrinogen are useful for this purpose.

In addition to the biomarkers mentioned above, there are others that have been evaluated in AF. Many have limited supporting evidence and require further studies to confirm their possible predictive capabilities. These include mean platelet volume, matrix metalloproteinase-2, reduced nicotinamide adenine dinucleotide phosphate oxidase 2-derived peptide, soluble CD40 ligand and tissue plasminogen activator (58, 60, 68, 70, 75, 78). There are also biomarkers that have not been thoroughly evaluated but thus far not been convincingly shown to be associated with stroke risk in AF. These include soluble fibrin monomer complex, antithrombin III, E-selectin, P-selectin, intercellular adhesion molecule-1, vascular cell adhesion molecule-1, prothrombin fragment F1+2, thrombin-antithrombin complexes, plasminogen activator inhibitor-1, and β-thromboglobulin (54, 58, 64, 68, 74, 76, 78, 79).

#### Urine Biomarkers

Few urine biomarkers have been identified as possible predictors of stroke risk in AF. In the ATRIA study, presence of proteinuria was associated with a 1.5-fold increased risk of thromboembolism (73). In addition, a retrospective study demonstrated that higher levels of albuminuria were associated with greater risk of thromboembolism among patients with newly diagnosed AF (104). Possible mechanisms are: (1) albuminuria reflects early-stage CKD which has been shown to be related to stroke risk; (2) albuminuria causes an imbalance between naturally occurring pro-thrombotic and anti-thrombotic factors, such as that seen in nephrotic syndrome (105). Pignatelli et al. showed that higher urinary prostaglandin F_2α_ levels were associated with a greater composite risk of stroke, TIA, myocardial infarction and coronary revascularisation during a follow-up period of 26 months (60). Furthermore, urinary 11-dehydro-thromboxane B2 has also been found to be related to a composite risk of stroke, TIA, myocardial infarction, coronary revascularisation, and cardiovascular-related death among patients with AF (106). Overall, although urine biomarkers have not been comprehensively investigated in AF, they may represent an additional, simple, and non-invasive method to aid stroke risk stratification.

#### Structural Biomarkers

Atrial fibrillation causes significant structural changes including atrial remodeling that may be detected through a variety of imaging techniques. Some of these changes have been found to predict stroke risk in AF (Table 4), potentially by promoting abnormal blood stasis. Early studies in this area have relied predominantly on standard transthoracic echocardiography. Recently, advanced imaging modalities with increased accuracy such as trans-esophageal echocardiography, computed tomography, and MRI have become more widely available. This has allowed the discovery of new structural biomarkers such as LA fibrosis and left atrial appendage (LAA) morphology that may be used to refine stroke risk assessment in AF. The SPAF study evaluated the role of 14 echocardiographic parameters to predict incident ischemic stroke or systemic embolism in AF (117). The authors reported that the presence of left ventricular dysfunction and higher LA size were found to be important. Furthermore, these parameters were able to identify patients without clinical risk factors who were at higher risk of stroke. A prospective study of 2,713 patients with AF demonstrated that LA enlargement (>45 mm) was linked to a 1.7-fold increased risk of ischemic stroke or systemic embolism (108). Dakay et al. also found that more severe LA enlargement was associated with a greater risk of ischemic stroke despite anticoagulation (107). Interestingly, there appears to be an association between LA size and stroke risk even in the absence of AF. In the Framingham Heart study, non-AF patients with increased LA size were found to be at greater risk of stroke and mortality during the follow-up period of 8 years (118). The presence of LA enlargement may therefore be helpful to identify the subset of AF patients who remain at high-risk of stroke despite anticoagulation therapy. Furthermore, it can be assessed on transthoracic echocardiography without the need for more complex imaging techniques.

**Table 4:** Structural biomarkers for stroke risk in AF.

**References**	**Design**	**Study population**	***n***	**% male; mean (SD) age, years**	**Relevant outcome measures**	**Follow-up duration**	**Biomarker**	**Findings**
Dakay et al. (107)	Prospective cohort	AF hospitalized with ischemic stroke	225	44.4; 79.5 (10.5)	Anticoagulation failure	NA	LAE	More severe left atrial enlargement was associated with greater risk of anticoagulation failure resulting in stroke
Hamatani et al. (108)	Prospective registry	AF	2,713	60.2; 73.7 (NA)	Ischemic stroke or SE	32.6 months	LAE	LA diameter >45 mm was associated with a 1.7-fold greater risk of ischemic stroke or SE
Kong et al. (109)	Prospective cohort	Drug-refractory AF undergoing catheter ablation	219	65.3; 58.1 (NA)	Stroke	NA (retrospective)	LAA morphology	Non-chicken wing morphology was associated with 5.8-fold greater stroke risk
Khurram et al. (110)	Prospective cohort	AF referred for catheter ablation	678	74.8; 59.5 (9.7)	Stroke or TIA	NA (retrospective)	LAA morphology	No association with stroke or TIA
							LAA trabeculations	Extensive LAA trabeculation was associated with a greater stroke or TIA risk
							LAA orifice diameter	Smaller LAA orifice was associated with a greater stroke or TIA risk
							LAA length	Shorter LAA length was associated with a greater stroke or TIA risk
Kimura et al. (111)	Case-controlled study	Drug-refractory AF who underwent catheter ablation	80	82.5; 58.6 (6.0)	Stroke	NA (retrospective)	LAA morphology	Cauliflower morphology was associated with a greater stroke risk
Di Biase et al. (112)	Prospective cohort	Drug-refractory AF undergoing catheter ablation	932	79; 59 (10)	Ischemic stroke or TIA	NA (retrospective)	LAA morphology	Chicken wing morphology was associated with lowest risk of ischemic stroke or TIA; with chicken wing morphology as reference, cactus, windsock and cauliflower were associated with a 4.1-, 4.5-, and 8.0-fold greater risks of ischemic stroke or TIA, respectively
Beinart et al. (113)	Case-controlled study	Non-anticoagulated AF	144	75; 54.5 (9.9)	Stroke or TIA	NA (retrospective)	LAA volume	No association with stroke or TIA
							LAA depth	No association with stroke or TIA
							LAA neck dimensions	High LAA neck dimension was associated with greater stroke or TIA risk
							LAA number of lobes	No association with stroke or TIA
Goldman et al. (114)	Sub-study of prospective cohort	AF with at least 1 high-risk stroke factor^*^	721	76; 68 (9)	Ischemic stroke or SE	NA	LAA peak antegrade flow velocity	LAA peak antegrade flow velocity <20 cm/s was associated with greater risk of Ischemic stroke or SE
Zabalgoitia et al. (115)	Sub-study of prospective cohort	AF with at least 1 high-risk stroke factor^*^	786	76; 69 (9)	Ischemic stroke or SE	NA	LAA thrombus	Presence of LAA thrombus was associated with a 2.5-fold greater risk of Ischemic stroke or SE
							SEC	Presence of SEC was associated with a 3.7-fold greater risk of Ischemic stroke or SE
							LAA peak antegrade flow velocity	LAA peak antegrade flow velocity <20 cm/s was associated with a 1.7-fold greater risk of Ischemic stroke or SE
							Complex aortic plaque	Presence of complex aortic plaque was associated with a 2.1-fold greater risk of Ischemic stroke or SE
Leung et al. (116)	Prospective cohort	AF undergoing TOE	272	68; 68 (11)	Stroke or SE	17.5 months	LA SEC	Presence of LA SEC was associated with a 3.5-fold greater risk of stroke or SE
SPAF (117)	Sub-study of RCT	AF	568	70; 67 (12)	Ischemic stroke or SE	1.3 years	14 echocardiographic parameters	LV dysfunction and higher LA size were the associated with greater risk of Ischemic stroke or SE

AF, atrial fibrillation; IQR, interquartile range; LA, left atrial; LAA, left atrial appendage; LAE, left atrial enlargement; LV, left ventricular; NA, not applicable or available; RCT, randomized controlled trial; SD, standard deviation; SE, systemic embolism; SEC, spontaneous echo contrast; TIA, transient ischemic attack; TOE, trans-esophageal echocardiography.

* *Similar study cohort*.

The majority of cardioembolic strokes originate from the left atrium. In the LA, the most common site of thrombus formation is within the LAA. This is a small, complex, pouch-like sac attached to the anterior portion of the LA. Due to its complex anatomical structure and narrow inlet, the LAA is prone to abnormal blood stasis predisposing to thrombus formation. These thrombi may subsequently dislodge to cause a stroke. Therefore, it is perhaps unsurprising that certain LAA features have been shown to influence stroke risk. Unfortunately, these are rarely appreciated on standard transthoracic echocardiography which remains the most commonly used imaging method. The SPAF-III study demonstrated that among AF patients, ongoing arrhythmia during trans-esophageal assessment was associated with a lower LAA peak antegrade flow velocity (Av_p_) (114). Furthermore, the authors found that an LAA Av_p_ <20 cm/s was related to the presence of spontaneous echo contrast and LAA thrombus, and increased risk of cardioembolic events. Predictors of Av_p_ <20 cm/s were increasing age, higher systolic blood pressure, ischemic heart disease, and greater LA area. All of which are known risk factors for AF. In addition, the presence of spontaneous echo contrast and LAA thrombus have both been independently shown to be linked to greater stroke risk in AF (115, 116).

It is now recognized that the LAA is a complex structure with significant variation between patients. A study by Di Biase et al. found that among AF patients planned for catheter ablation, LAA morphologies could be categorized into four main groups based on their appearances on computed tomography or MRI (112). In order of reducing frequency, these were called “chicken wing” (48%), “cactus” (30%), “windsock” (19%), and “cauliflower” (3%). After multivariable adjustment, a chicken wing morphology was associated with the lowest risk of stroke. In comparison, there was a 4-fold increased stroke risk with the cactus and windsock morphologies, and 8-fold increased stroke risk with the cauliflower morphology. Similar findings were also reported elsewhere (109, 111). However, a study by Khurram et al. failed to demonstrate any association between LAA morphology and risk of stroke or TIA (110). In addition, the authors found that there was significant inter-observer variability during determination of LAA morphology, indicating that this may be an unreliable method of assessment. Limitations of the studies assessing LAA morphology above lies in the fact that they were all retrospective in nature and included only a subset of AF patients, specifically those undergoing catheter ablation. Therefore, future prospective studies are needed to confirm whether LAA morphology may be used for stroke risk stratification in a general cohort of AF patients.

Other LAA parameters such as the number of lobes, neck dimension, overall dimension, volume, orifice diameter, and trabeculations have also been studied but again further evaluation is required (110, 113). Left atrial fibrosis may also represent an additional biomarker for stroke risk stratification. In a study of 178 patients with AF, LA fibrosis was assessed using late gadolinium enhancement MRI and correlated to trans-esophageal findings (119). The authors reported that high atrial fibrosis (>20%) was linked to spontaneous echo contrast and LAA thrombus. Additionally, the presence of complex aortic plaques on trans-esophageal echocardiography defined based on features of mobility, ulceration, pedunculation, thickness ≥4 mm, and location were found to be independently associated with a 2-fold increased thromboembolic risk (115).

### Genetic Markers

Improvements in genomic technologies have seen an increasing role for genetic testing in certain diseases. This may provide an additional element for risk stratification in AF. However, there have been few genetics studies in AF to date and they have largely focused on chromosome 4q25. It has been suggested that genetic variants on this chromosome may be related with ischemic stroke (120, 121). In a case-control study of 1,059 AF patients, after adjusting for potential confounders, FGB 455 G/A polymorphism was associated with increased cardioembolic stroke potentially through elevated fibrinogen levels (122). Factor V Leiden mutation has not been found to be predictive of thromboembolism in AF (79). Overall, more studies are needed to confirm these genetic findings. Even then, the use of genetic markers for stroke risk stratification in AF remains unrealistic at present.

## Bleeding Risk Assessment

A vital aspect of the management for AF includes stroke prevention. To this end, many patients require anticoagulation therapy. However, this approach is not without risk. A meta-analysis of eight RCTs found that the annual rates of major bleeding varied from 1.4 to 3.4% among patients with AF treated with warfarin (123). The risk of ICH, the most serious form of bleeding, was estimated at 0.61% per year. Similar results were reported by Fang et al. in a cohort of 13,559 patients with AF treated with warfarin (124). Despite the relatively low rates of ICH, 76% of these patients had severe disability or died, and ICH was associated with at least a 20-fold increased risk of 30-day mortality compared to other forms of bleeding. Given the detrimental consequences of anticoagulation-related bleeding in AF, especially with ICH, efforts should be directed at reducing this risk while maintaining adequate stroke prevention. The use of NOACs have been shown to be superior to warfarin in this regard. Two large meta-analysis have shown that NOACs, as a class of medications, have a better safety profile with less major bleeding and ICH when compared to warfarin (125, 126).

It is also important to consider the timing of anticoagulation-related bleeding events. In this aspect, there appears to be an excess risk during the initial few months of treatment with vitamin K antagonist (VKA) (127). This may be due to poor anticoagulation control that eventually improves with time. However, there are likely to be additional factors involved as a similar effect was observed with dabigatran, where initial dose adjustments are rare (128). It is possible that the use of anticoagulation is simply unmasking high-risk individuals who were not identified using traditional assessment methods. Therefore, better risk profiling is necessary. Various factors based on clinical, biological, and genetic markers have been shown to predict the risk of anticoagulation-related bleeding in patients with AF (Table 5). Some of these factors may also influence the stroke risk.

**Table 5:** Risk factors for anticoagulation-related bleeding.

	**Risk factor**	**Possible risk factor**
Clinical markers	History of bleeding Antiplatelets or NSAID use Excess alcohol Uncontrolled hypertension Increasing age Malignancy Prior stroke Vascular disease Race/ethnicity (non-white) Choice of anticoagulant	Diabetes mellitus Female sex Prior falls Thyroid disease Prior MI or known IHD
Biological markers	Poor anticoagulation control (high INR or reduced TTR) Liver dysfunction Renal dysfunction Anemia Reduced platelet count or function	Interleukin-6 von Willebrand factor Growth differentiation factor-15 Troponins
Genetic marker	CYP 2C9 polymorphism	

*CYP, cytochrome P450; IHD, ischemic heart disease; INR, international normalized ratio; MI, myocardial infarction; NSAID, non-steroidal anti-inflammatory drug; TTR, time-in-therapeutic range*.

There are several bleeding risk scores designed specifically for use in an AF cohort (Table 6). They have previously been summarized and include a combination of different clinical, biological and genetic markers (132). In general, each risk factor is assigned a score and the sum of these scores are used to estimate annual bleeding risk in an individual who is anticoagulated. It should be noted that there are differences in the way certain risk factors (e.g., age, renal dysfunction, and hypertension) have been defined between the various risk scores. Furthermore, many risk factors for bleeding contribute as well to stroke risk in AF. This highlights the complex relationship between thrombogenesis and bleeding, and represent the challenges faced by physicians when weighing up the risk and benefits of anticoagulation therapy (20).

**Table 6:** Bleeding risk scores in AF.

**Risk factors**	**ABC-bleeding (129)**	**ATRIA bleeding (130)**	**HAS-BLED (18)**	**HEMORR _**2**_HAGES (131)**	**ORBIT (131)**
Antiplatelets or NSAID use			x		x
Diabetes mellitus					
Excess alcohol			x	x	
Excessive falls risk				x	
Females					x
History of bleeding	x	x	x	x	x
Hypertension		x	x	x	
Elderly patients	x	x	x	x	x
Malignancy				x	
Previous stroke			x	x	
Abnormal liver function			x	x	
Abnormal renal function		x	x	x	x
Anemia	x	x		x	x
Labile INR (TTR <60%)			x		
Raised GDF-15	x				
Raised hsTrop	x				
Reduced platelet count or function				x	
CYP 2C9 polymorphism				x	
Total score	45	10	9	12	7

*CYP, cytochrome P450; GDF-15, growth differentiation factor-15; hsTrop, high-sensitivity troponin; INR, international normalized ratio; NSAID, non-steroidal anti-inflammatory drug; TTR, time-in-therapeutic range*.

### Clinical Markers

An early study investigating the risk factors for bleeding among patients treated with warfarin found that age ≥65 years, prior stroke, history of gastrointestinal bleeding, presence of serious comorbidity (such as recent myocardial infarction or renal impairment) and AF were important predictors (133). However, the study was limited by a small sample size and heterogenous cohort. Hughes et al. performed a systematic review of nine studies reporting on anticoagulation-related bleeding complications in AF to demonstrate that increasing age, uncontrolled hypertension, prior myocardial infarction or ischemic heart disease, prior stroke, anemia, history of bleeding, and concomitant use of other drugs (e.g., antiplatelets) were independent risk factors for bleeding (134). Unlike previous studies (135, 136), diabetes mellitus and sex were not found to be important predictors. Conversely, age and concomitant use of antiplatelets have been consistently shown to significantly increase the risk of anticoagulation-related major bleeding (129, 135–137). Potential explanations for the increased risk of bleeding with age may relate to changes in metabolic clearance, higher prevalence of comorbidities, degenerative vascular changes, polypharmacy, and cognitive decline (138). Meanwhile, concomitant use of antiplatelets will interfere with additional hemostatic pathways that are necessary to prevent bleeding. Additional anticoagulation-related bleeding risk factors that have previously been described include excess alcohol intake and thyroid disease (136, 139).

#### Falls

Prior falls is perceived as a risk factor for anticoagulation-related bleeding, especially in elderly patients. Gage et al. demonstrated a significantly increased risk of ICH associated with the use of warfarin in AF patients deemed at high-risk of falls (140). Based on this, such patients are often deprived of anticoagulant treatment for stroke prevention with the assumption that it is harmful. However, it is important to recognize that within the same study, there was overall improvement in clinical outcomes among patients at high-risk of falls who received anticoagulation therapy, despite the increased risk of ICH. In a separate study involving 7,156 patients with AF, a history of falls was independently associated with a 3.3-fold increased risk of major bleeding (141). The authors remarked that assessment of “actual falls” may be more clinically useful than the “falls risk.” This may provide an explanation for lack of association between falls and major bleeding events in a prospective study involving 515 patients on oral anticoagulation, where falls risk was used (142). Overall, the current evidence indicate that a history of falls may be an important risk factor for major bleeding but that this should not be the sole deterrent for anticoagulation in AF. In support of this, an earlier study by Man-Son-Hing et al. found that patients would need to fall an estimated 295 times per year for the risk of serious bleeding to outweigh the beneficial effects of warfarin (143).

#### Malignancy

Presence of a malignant disease has been associated with increased anticoagulation-related bleeding. A study by Gitter et al. found that patients with a malignant condition at the time of warfarin initiation had a 4-fold greater risk of major hemorrhage during a 28-month follow-up period (144). Results from a secondary analysis of a prospective RCT demonstrated that the higher risk of bleeding was also observed with other anticoagulants such as heparin and danaparoid (145). Despite the overall increased risk of anticoagulation-related bleeding with malignant conditions, there exist variations in terms of safety and efficacy between the different options for anticoagulation. In general, low-molecular weight heparin has a similar safety profile to VKA and is superior for prevention of recurrent thromboembolism (146, 147). Non-vitamin K antagonist oral anticoagulants are a more convenient option to low-molecular weight heparin but may be associated with a higher risk of major bleeding with comparable effectiveness (147–149). Therefore, patients with malignant conditions who require anticoagulation therapy should be commenced on either low-molecular weight heparin or NOACs.

#### Ethnicity

A previous study of 28,628 patients from the GARFIELD-AF registry demonstrated that those with an Asian background had reduced risk of major bleeding compared to other ethnicities (150). However, subsequent studies have found that while the risk of ICH is higher among non-whites in general, it was up to 4-fold higher in Asians compared to whites (151, 152). Furthermore, the complication was associated with worse outcomes in this group of patients (153). Given the current evidence, additional attention should be directed toward other modifiable bleeding risk factors in Asian patients commencing anticoagulation therapy. It may also be appropriate to consider NOACs in the first-instance as these have been found to be safer alternatives to VKA in this cohort (154).

#### Weight

Body weight is known to influence the distribution and clearance of anticoagulants (155). Therefore, there were concerns about the safety (and efficacy) of these medications in patients with extremes of body weight. However, studies on this topic have produced reassuring results. The risk of anticoagulation-related bleeding appears similar in underweight, and overweight or obese patients compared to those with normal body weight (156, 157). In fact, some studies have suggested that obesity may even be associated with reduced rates of bleeding (158–160). Furthermore, patients with obesity and AF who were treated with a NOAC had lower risk of thromboembolism, leading to the term “obesity paradox” (156–158).

### Biological Markers

There are several biomarkers that may help predict anticoagulation-related bleeding. Some of these are linked to stroke risk and have already been described above (e.g., renal failure, IL-6, vWF). Perhaps one of the most important biomarkers associated with the use of VKA is the intensity of anticoagulation, measured as the international normalized ratio (INR). Higher INR levels have been found to increase the risk of anticoagulation-related bleeding, with relative risks (RR) for INR ≥4.5 of 7.9 (95% CI, 5.4–11.5) compared to INR <4.5 (127). It is estimated that every one unit increase in INR above 2.5 was associated with a 2-fold increased risk of mortality (161). Therefore, every effort should be taken to maintain the INR of AF patients within pre-defined ranges, often between 2 and 3 (20). However, absolute INR levels alone can be misleading as a significant proportion of major bleeds occur despite the INR being in therapeutic range (162). More recently, INR variability has emerged as a more reliable assessment method for bleeding risk. A “labile INR” as determined using time-in-therapeutic range (TTR) is strongly linked to future bleeding events (163–165). However, there are several limitations of TTR that are worth bearing in mind (166, 167). Firstly, it assumes a linear relationship between INR measurements which may not be true. Secondly, it does not inform on short-term risks associated with extreme deviations in INR. Lastly, it fails to account for individuals with “missed” monitoring periods who may represent a group at higher risk of bleeding due to reasons such as non-adherence.

Given the importance of the liver and kidneys in regulating pharmacokinetics of drugs and maintaining hemostasis, it is inevitable that fluctuations in the functions of these organs could negatively impact on anticoagulation-related bleeding. A prospective, observational study of 8,466 AF patients treated with either VKA or NOACs suggests that both abnormal renal and liver functions were associated with increased risk of major bleeding (168). The study authors defined abnormal renal function as serum creatinine >2.3 mg/dL (200 μmol/L), prior renal transplantation or receiving chronic dialysis, and abnormal liver function as cirrhosis, elevated liver transaminases, or alkaline phosphatase >3 times above the upper limit of normal, or bilirubin >2 times above the upper limit of normal. Similar findings were reported in a separate large cohort study of 7,141 AF patients receiving rivaroxaban (169). Furthermore, Banerjee et al. reported that lower levels of eGFR were related to a greater risk of major bleeding over a study period of 2.5 years (61).

Anemia has also been linked to increased bleeding risk (133, 134, 170). The underlying mechanism remains unclear but there is some evidence to suggest that platelet aggregation is impaired by a reduced red blood cell count (171). Furthermore, the anemia may indicate concealed bleeding that becomes manifest with anticoagulation therapy.

The use of IL-6 as a predictor of anticoagulation-related bleeding remains controversial. A large cohort study in AF patients found that IL-6 was independently associated with bleeding following adjustment for clinical risk factors and other biomarkers (troponin, NT-proBNP, and cystatin-C) (59). Meanwhile, although a separate large cohort study initially demonstrated the relationship between IL-6 and bleeding, the results were not statistically significant once other biomarkers were taken into account (172). Neither of these studies found CRP to be important in the relationship with bleeding.

Similar to the stroke findings as discussed above, the study by Janion-Sadowska et al. found no association between thrombocytopenia (platelet count <100 × 10^9^/L) and risk of bleeding (53). In contrast, Park et al. reported that patients with a platelet count <100 × 10^9^/L had a significantly increased risk of bleeding events compared to those with a normal platelet count [HR 2.19 (95% CI, 1.77–2.70)] (52). Despite limited evidence on the matter, it seems likely that a low platelet count could increase the risk of bleeding in AF.

In addition to the biomarkers above, there are several others that have been associated with anticoagulation-related bleeding including vWF, high-sensitivity troponin, and growth differentiation factor-15 (marker of oxidative stress). Roldan et al. showed that patients with high levels of vWF had a 4.5-fold increased risk of major bleeding (69). Using the ARISTOTLE cohort, Hijazi et al. demonstrated that both high-sensitivity troponin and growth differentiation factor-15 were the strongest predictors of major bleeding when compared to traditional risk factors such as age, hemoglobin, previous bleeding, congestive heart failure, previous stroke or TIA, hypertension, and diabetes mellitus (129). Studies on NT-proBNP have not found it to be useful for predicting anticoagulation-related bleeding (62, 65, 66). Overall, there is limited research to support the “real world” role of these biomarkers in relation to bleeding risk assessment, given that bleeding risk is dynamic and changes with addressing modifiable risks, and that many biomarkers are non-specific and likely to reflect a sick patient or “sick heart.”

### Genetic Markers

Polymorphism of cytochrome P450 2C9 has been linked to an increased risk of major bleeding through its effects on the metabolism and action of warfarin (173). It may also have important implications on warfarin dose requirements (174). However, as mentioned above, there is currently limited evidence on the role for genetic markers in AF.

## Limitations of Risk Scores

Risk scores are useful as they provide a rapid tool to guide treatment decisions in AF and highlight bleeding risk factors that deserve attention. However, it is important to recognize that the risk scores in AF have been simplified to provide physicians with a reliable yet useable tool for daily clinical practice. As a result, most are subject to several limitations and are at best, only modestly robust at predicting individual stroke risk (175).

First, not all risk factors may be included in certain risk scores. Second, they use a “one size fits all” approach and do not account for the heterogenous nature within the AF population. Third, they fail to adequately consider the differential weight of individual risk factors. Fourth, they fail to consider the degree or severity of individual risk factors. Fifth, many risk scores were developed using older definition of diseases that may have subsequently evolved over time. Finally, studies have often correlated stroke occurrences during long periods of follow-up to risk factors measured at baseline, and risk changes with increasing age and incident risk factors. Indeed, recent attention has been directed toward the dynamic nature of risk profiles in AF patients (176–178). Chao et al. found the majority of patients with AF (89.4%) developed ≥1 new risk factor(s) prior to presenting with an ischemic stroke (178). Indeed, a change in CHA_2_DS_2_-VASc score was demonstrated to be strongly predictive of ischemic stroke. The study highlights the importance of regular stroke risk assessments in AF. Therefore, stroke and bleeding risk stratification should be undertaken by clinicians as a continuous process with specific focus on preventing the development of additional risk factors. Furthermore, in addition to the dynamic nature of risk, therapeutic options for AF are expanding. As more effective and safer therapies are introduced, we may need to re-evaluate the threshold for initiating anticoagulation.

## Conclusion

In conclusion, there are a variety of clinical, electrical, biological, and genetic markers to guide stroke and bleeding risk assessments in AF. Furthermore, risk schemas provide a structured, standardized, and rapid tool for this purpose.

## Author Contributions

WD drafted the manuscript. SH, DG, DL, and GL provided critical appraisal. All authors approved the final version of the manuscript and agree to be accountable for the accuracy or integrity of the manuscript.

### Conflict of Interest

DG: Speaker for Bayer, BMS/Pfizer, Boehringer Ingelheim, Daiichi-Sankyo, Medtronic, Biosense Webster, and Boston Scientific. Proctor for Abbott. Research Grants from Medtronic, Biosense Webster, and Boston Scientific. GL: Consultant for Bayer/Janssen, BMS/Pfizer, Medtronic, Boehringer Ingelheim, Novartis, Verseon, and Daiichi-Sankyo. Speaker for Bayer, BMS/Pfizer, Medtronic, Boehringer Ingelheim, and Daiichi-Sankyo. No fees are directly received personally. DL has received investigator-initiated educational grants from Bristol-Myers Squibb and Boehringer Ingelheim; has been a speaker for Boehringer Ingelheim, Bayer, and Bristol-Myers Squibb/Pfizer; and has consulted for Bristol-Myers Squibb, Bayer, Boehringer Ingelheim, and Daiichi-Sankyo. The authors declare that the research was conducted in the absence of any commercial or financial relationships that could be construed as a potential conflict of interest.
